# Flow of CO_2_ from soil may not correspond with CO_2_ concentration in soil

**DOI:** 10.1038/s41598-018-28225-z

**Published:** 2018-07-04

**Authors:** Jan Frouz, Luděk Bujalský

**Affiliations:** Institute for Environmental Studies, Faculty of Sciences & Environmental Centre, Charles University, Benátská 2, 12800 Prague, Czech Republic

## Abstract

The relationship between CO_2_ flow from soil and soil CO_2_ concentration was investigated at 72 permanent sampling points at two forested post-mining sites in the northwest of the Czechia. Based on the entire data set (72 points sampled monthly during the growing season), CO_2_ flow from the soil was positively correlated with soil CO_2_ concentration. CO_2_ concentration in deeper soil layers was positively correlated with root biomass and negatively correlated with soil microbial respiration. In individual sampling points relationship between CO_2_ flow and soil CO_2_ concentration varied from being significantly positive (30% of points) to significantly negative (7%) but mostly being non-significant (63%). The positive correlation occurred at points with high root biomass in deeper soil layers, while the negative correlation occurred at points with high soil microbial respiration per cm^3^ of soil. Laboratory experiments showed that the CO_2_ produced by microbial respiration can reduce microbial respiration but that CO_2_ produced by root respiration did not reduce root respiration. The results indicate that when soil ventilation is poor, microbial respiration can sufficiently increase soil CO_2_ concentration so as to reduce microbial respiration, which greatly increases the variability in the relationship between CO_2_ flow from soil and soil CO_2_ concentration.

## Introduction

The soil is an important global carbon (C) pool because it contains two- to three-times more C than the atmosphere^1^. The CO_2_ flux from the soil (i.e., soil respiration) represents an important part of the global C cycle because it supplies most of the CO_2_ that moves from terrestrial ecosystems to the atmosphere^2,3^. The mean annual soil respiration was reported to be 49% of the gross primary production in 18 forest sites of the EUROFLUX project^4^. Soil respiration can be a sensitive indicator of the overall soil metabolic activity and can be used as a measure of soil recovery at reclaimed sites^5^. Soil respiration has two main components: heterotrophic respiration (based on the decomposition and mineralization of soil organic matter, largely by microorganisms) and autotrophic or root respiration^6^. The proportion of heterotrophic and root respiration may vary substantially because root respiration can represent from 10 to 90% of the total soil respiration^6^. Factors affecting soil respiration are complex and include soil temperature, moisture, C availability, and the relative contribution of root respiration^3,7,8^.

Soil respiration can be measured by placing various kinds of chambers (static or dynamic) on the soil surface^9,10^ or by measuring the CO_2_ concentration at various depths in the soil profile and calculating the CO_2_ flux based on diffusion theory^11–16^. The profile method calculates CO_2_ flux based on Fick’s first law, which uses the vertical gradient of the soil CO_2_ concentration and the gaseous diffusion coefficient of soil CO_2_. The profile method does not consider consumption of CO_2_ or any kind of negative feedback loop in which CO_2_ production would decrease as the soil CO_2_ concentration increases. Studies have shown, however, that in alkaline soils CO_2_ can be absorbed in the soil in such extend that in extreme causes result in negative flow of CO_2_ from the soil^17^. Moreover, soil microbes can metabolize CO_2_ via “heterotrophic fixation”^1,18^ and that an increase in soil CO_2_ concentration can reduce microbial respiration and thus CO_2_ input to the soil^19^. If these mechanisms are quantitatively important, they may cause substantial differences among soil “spots” (by spot, we mean the area assessed by a CO_2_ sensor as described in the Methods) in the relationship between the CO_2_ flux from the soil and the soil CO_2_ concentration and may even cause the relationship to be negative namely in case when soil diffusion reduces (e.g. as consequence of increased moisture) microbes in poorly ventilated soil may produce enough CO_2_ to stop their own respiration. In such cases the concentration will remain high but flow will be reduced. By other words such negative feedback loop between CO_2_ production and soil CO_2_ concentration may substantially enhance variation between CO_2_ flow and soil CO_2_ concentration given by natural variation in diffusion coefficient.

**Figure 1:**
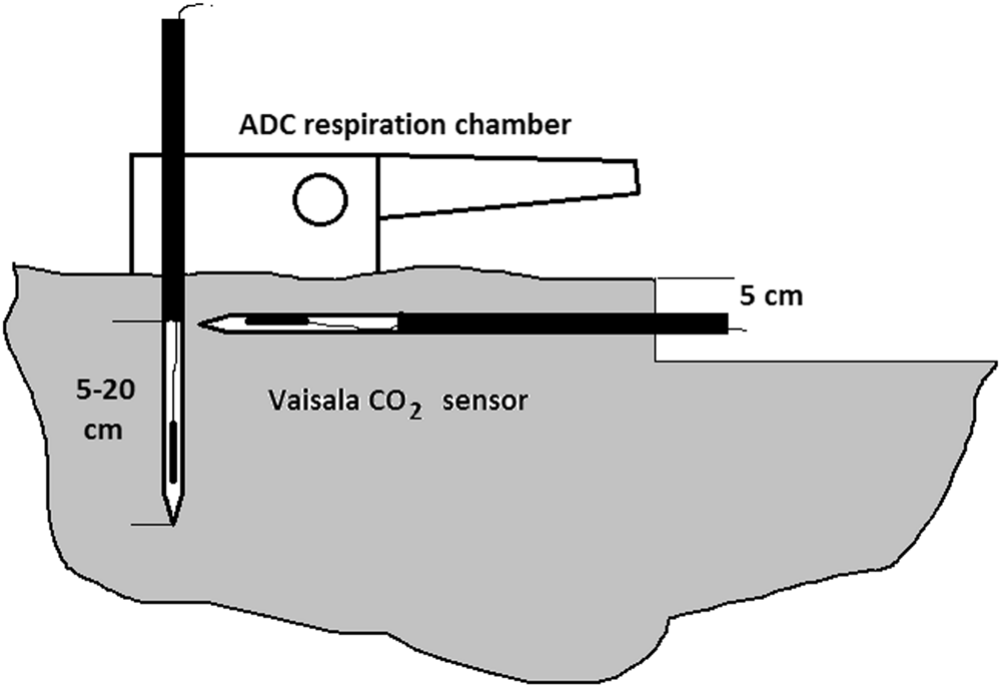
Diagram of how CO_2_ flow from soil and CO_2_ concentration in soil were measured at one sampling point in the field. At each sampling point, CO_2_ flow from the soil was measured with an ADC respiration chamber, and soil CO_2_ concentration was measured with two Vaisala sensors (one at 5 cm depth and one at 5–20 cm depth).

**Figure 2:**
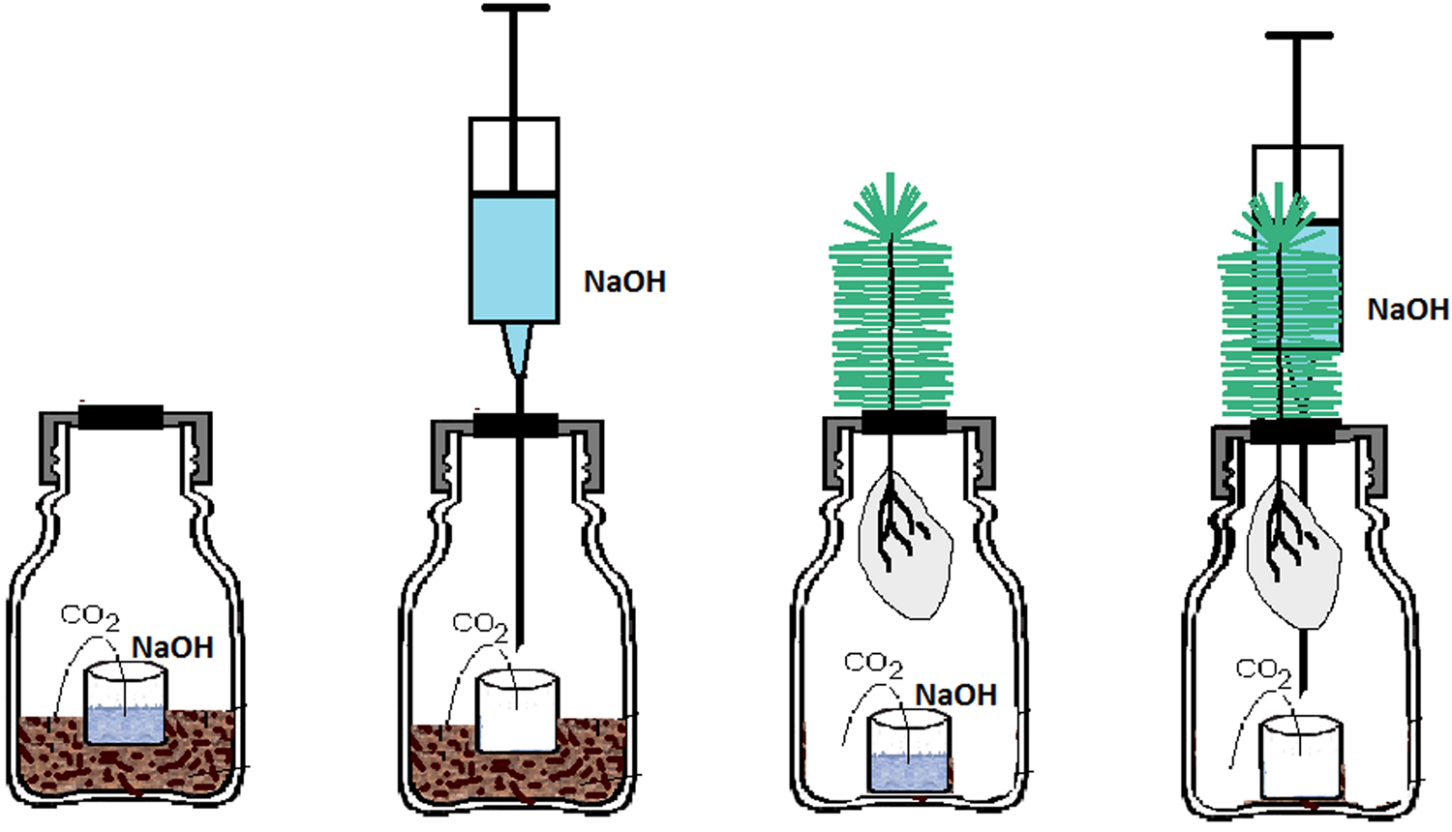
Diagrams of the sealed vials and other equipment used in the two laboratory experiments in which CO_2_ was “ventilated” (by placing NaOH in the vial at the start of the incubation) or was not ventilated (by not placing NaOH in the vial until the end of the incubation). In both cases, respiration was measured by trapping the CO_2_ in NaOH, followed by titration. In the first laboratory experiment (the two diagrams on the left), the vials contained field soil. In the second laboratory experiment (the two diagrams on the right), the vials contained the roots of spruce seedlings in sand wrapped in nylon mesh. The experiments assessed whether there was negative feedback between microbial respiration and soil CO_2_ concentration or between root respiration and soil CO_2_ concentration. Low and high soil CO_2_ concentrations were generated in the ventilated and non-ventilated vials, respectively.

In the current study, we examined the relationship between the CO_2_ flux from the soil and the CO_2_ concentration in the soil (Fig. 1). We test the hypothesis that although the overall relationship between CO_2_ flux and CO_2_ concentration will be positive, the relationship among local spots will vary from highly positive to highly negative. We also expect that negative relationships will be more common in spots where soil respiration is dominated by microbes rather than by roots. To test these hypotheses, we used field sites that are known to have a large variation in the contributions of microbes and roots to the overall respiration but whose other parameters are very similar^20–25^. We also conducted laboratory experiments with soil from the same field sites and with one of the sub-dominant plant species to test the hypothesis that microbes but not roots are likely to increase the CO_2_ concentration to the level that reduces their own respiration (Fig. 2).

## Results

**Figure 3:**
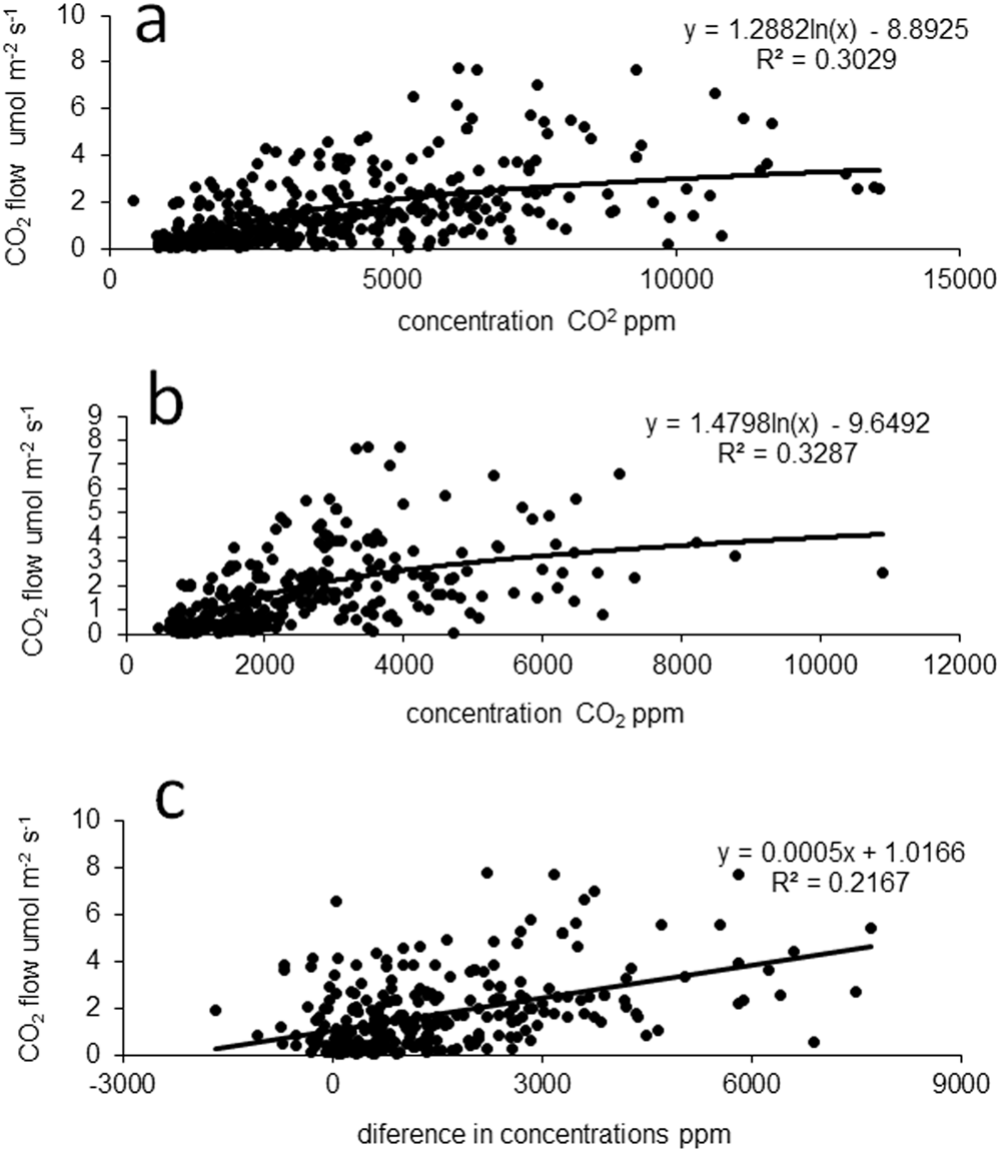
Relationships between CO_2_ flow from the soil and (**a**) soil CO_2_ concentration at 5–20 cm depth, (**b**) soil CO_2_ concentration at 5 cm depth, and (**c**) the difference between the soil CO_2_ concentration at the two depths. Each plot includes 576 values (36 sampling points per site × 2 sites × 8 sampling times).

Based on the whole dataset (i.e., data from both the reclaimed and unreclaimed site) and as indicated by regression using log-transformed values for soil CO_2_ concentration, CO_2_ flow from the soil was positively related to soil CO_2_ concentration at both depths (Fig. 3a,b). CO_2_ flow from the soil was also positively related to the difference in CO_2_ concentration between the deeper and shallower soil layers (Fig. 3c). Similar relationships were evident based on correlation analysis, i.e., CO_2_ flow was positively correlated with CO_2_ concentration at each depth across both sites and at each depth at each site (Table 1). Similarly, CO_2_ flow was positively correlated with the difference in CO_2_ concentration between the deeper and shallower layers across the two sites and at each site (Table 1).

**Table 1:** Pearson correlation coefficients between the soil CO_2_ concentration measured vertically in the 5–20 cm depth and horizontally in the 5 cm depth, the difference between these two concentrations, the CO_2_ flow from the soil, and other soil properties.

Soil property	CO_2_ flow	Temperature	Root biomass			Respiration (g^−1^)			Respiration (cm^−3^)		Microbial biomass			Porosity		
**Depth**			0–5 cm	5–10 cm	10–20 cm	0–5 cm	5–10 cm	10–20 cm	0–5 cm	5–10 cm	0–5 cm	5–10 cm	10–20 cm	0–5 cm	5–10 cm	10–20 cm
**All data**																
CO_2_ concentration (5–20 cm)	0.554	0.151		0.232	0.191		−0.133		−0.202				0.168		0.280	0.207
CO_2_ concentration (5 cm)	0.524			0.324	0.208		−0.130		−0.205				0.135	−0.121	0.316	0.198
Difference	0.466			0.124	0.183		−0.113		−0.180				0.152		0.211	0.226
CO_2_ flow		0.173		0.154	0.158		−0.125		−0.195						0.266	0.209
**Unreclaimed only**																
CO_2_ concentration (5–20 cm)	0.408										0.329	0.171				−0.142
CO_2_ concentration (5 cm)	0.496			0.147							0.240	0.231				
Difference	0.287	0.241		0.016												
CO_2_ flow			0.241	0.239				0.196		0.203	0.225		−0.206		0.211	
**Reclaimed only**																
CO_2_ concentration (5–20 cm)	0.445	0.432	0.171	0.242	0.141						0.152		0.227			
CO_2_ concentration (5 cm)	0.217	0.206	0.154	0.421							0.150		0.213	0.182		−0.172
Difference	0.321		0.193		0.167	−0.144							0.200			
CO_2_ flow		0.402											0.198	−0.146		

Non-significant coefficients are not shown.

Based on data from both sites, soil CO_2_ concentration was positively correlated with root biomass at 5–10 and 10–20 cm depths, with microbial biomass at 10–20 cm depth, and with porosity at 5–10 and 10–20 cm depths, but was negatively correlated with soil respiration (whether the latter was expressed per g of soil or per cm^3^ of soil) as determined in the laboratory incubation (Table 1). In addition to being correlated with soil CO_2_ concentration, CO_2_ flow from the soil (based on data from both sites) was positively correlated with soil temperature, root biomass at 5–10 and 10–20 cm depths, microbial biomass at 10–20 cm depth, and porosity at 5–10 and 10–20 cm depths, and was negatively correlated with soil respiration (whether soil respiration was expressed per g of soil or per cm^3^ of soil).

According to multiple regression, CO_2_ flow from the soil was significantly related to the CO_2_ concentration at the 5–20 cm depth, temperature, soil porosity at the 5–10 cm depth, and microbial respiration at the 5–10 cm depth (Table 2).

**Table 2:** Summary of multiple regressions describing the relationship between CO_2_ flow from the soil (dependent parameters) and other soil properties (predictor- regression parameters) (R^2^ = 0.40305916, F_7,288_ = 27.780, P < 0.0000).

Multiple regression parameter	Coefficient	P
absolute member	1.1232	0.0945
CO_2_ concentration (5–20 cm)	0.0003	0.0000
temperature	−0.0852	0.0083
porosity (5–10 cm)	1.3902	0.0476
respiration (5–10 cm)	−0.0611	0.0453
porosity (0–5 cm)	−0.0597	0.1691
microbial biomass (5–10 cm)	−0.0242	0.2661
porosity (10–20 cm)	0.5060	0.2995

**Figure 4:**
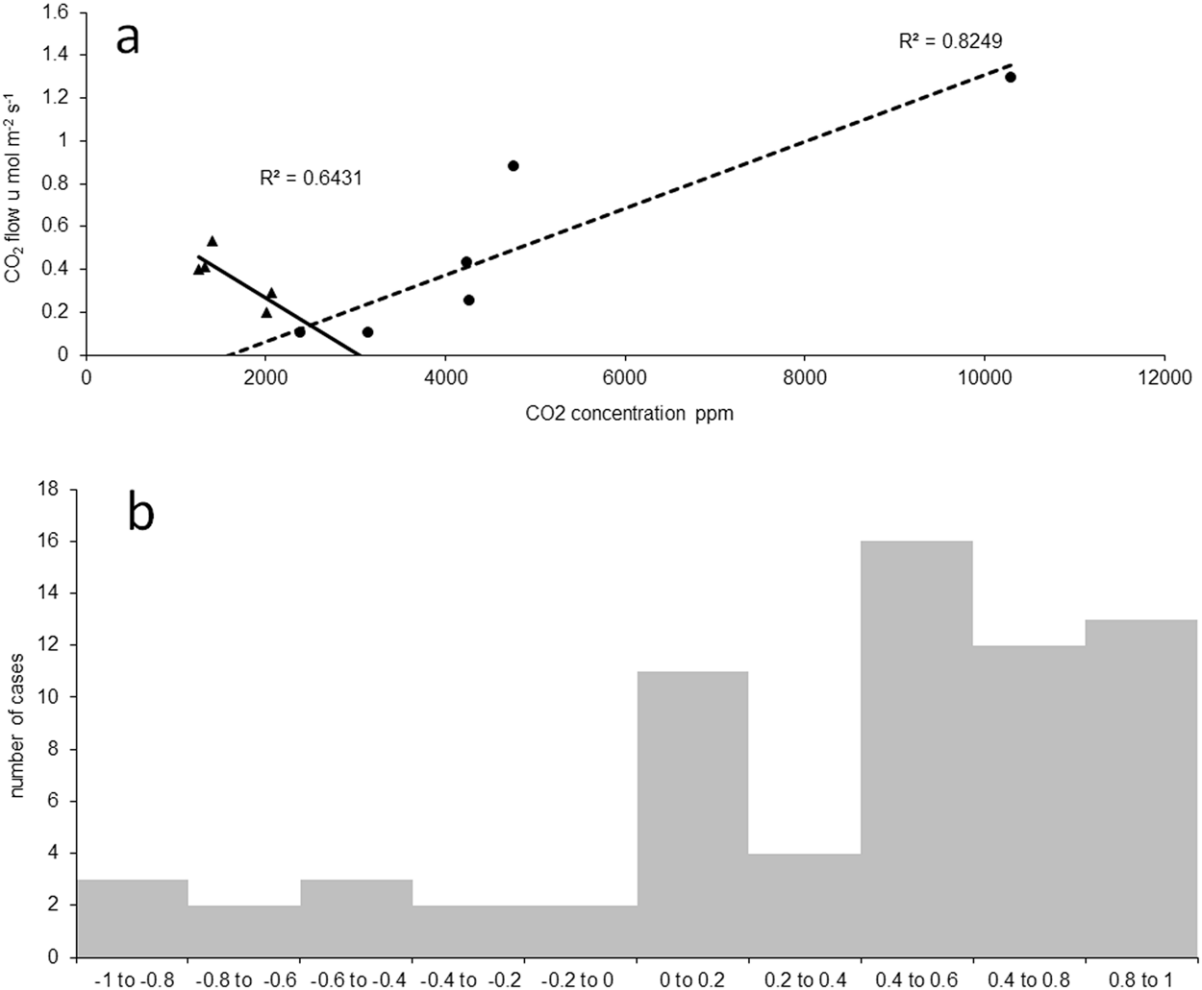
Example of the relationship between the CO_2_ flow from the soil and the soil CO_2_ concentration measured vertically in the 5–20 cm depth for two individual sampling points; individual measurements taken at different time at one point are marked by circles, for other points, individual measurements taken at different time are marked by triangles (**a**) and the frequency distribution of correlation coefficients for the relationship between CO_2_ flow from the soil and the soil CO_2_ concentration at 5–20 cm depth for all 72 sampling points (**b**).

To this point of the Results, we have evaluated the data across all points at one site or at both sites such that the variation in any parameter included both spatial variation between individual sampling points as well as temporal variation in individual sampling points. An examination of individual measurements at individual points (i.e., an analysis of the temporal change in the relationship as indicated by 9 samples collected at different times at each point) revealed that the relationship between CO_2_ flow from the soil and soil CO_2_ concentration may vary from highly positive (as indicated by the filled circles in Fig. 4a) to highly negative (as indicated by the filled triangles in Fig. 4a). A similar pattern of variation was found for the correlation coefficient (Fig. 4b) as well as for the slope of regression (Table 3). For 30% of the points, CO_2_ flow from the soil was significantly related to soil CO_2_ concentration, and the slope was positive. For about 7% of the points, CO_2_ flow from the soil was also significantly related to soil CO_2_ concentration but the slope was negative. For 63% of the points, the correlation was not statistically significant both positive and negative.

**Table 3:** Linear regression for dependent variables, regression coefficients, and slopes of the regression between the CO_2_ flow from the soil and the soil CO_2_ concentration at individual sampling points as depicted in Fig. 4 and environmental parameters measured at these sampling points.

Environmental parameters	r	Slope
CO_2_ concentration	0.29642	0.2668
root biomass (10–20 cm)		0.265033
bulk density (10–20 cm)		0.247234
	—	
respiration (cm^−3^ soil)	0.23199	

In a further analysis, we examined the relationships between the correlation coefficients and regression slopes for regressions between the soil CO_2_ concentration and the CO_2_ flow from the soil at each of the 72 measuring points (as described in the previous paragraph) and environmental conditions at these points. The average soil CO_2_ concentration at the 5–20 cm depth, root biomass at the 10–20 cm depth, and bulk density at the 10–20 cm depth were positively correlated with either the slopes, the correlation coefficients, or both the slopes and correlation coefficients between the CO_2_ flow from the soil and the soil CO_2_ concentration (Table 3). In contrast, soil respiration expressed per soil volume was negatively correlated with the correlation coefficient between the CO_2_ flow from the soil and the soil CO_2_ concentration. The negative and positive correlation coefficients and negative and positive regression slopes between the CO_2_ flow from the soil and the soil CO_2_ concentration were evenly distributed between both reclaimed and unreclaimed sites (the Pearson correlation coefficient between presence of reclamation and correlation coefficient or slope of regression for concentration flow relationship was 0.065 and 0.006 respectively). In other words, it seems that at least in some cases the relationship between the CO_2_ flow from the soil and the soil CO_2_ concentration is consistent with our second hypothesis (that microbes but not roots are likely to increase the CO_2_ concentration to a level that results in negative feedback on respiration) and that this is more likely to occur at points with high microbial respiration per soil volume.

**Figure 5:**
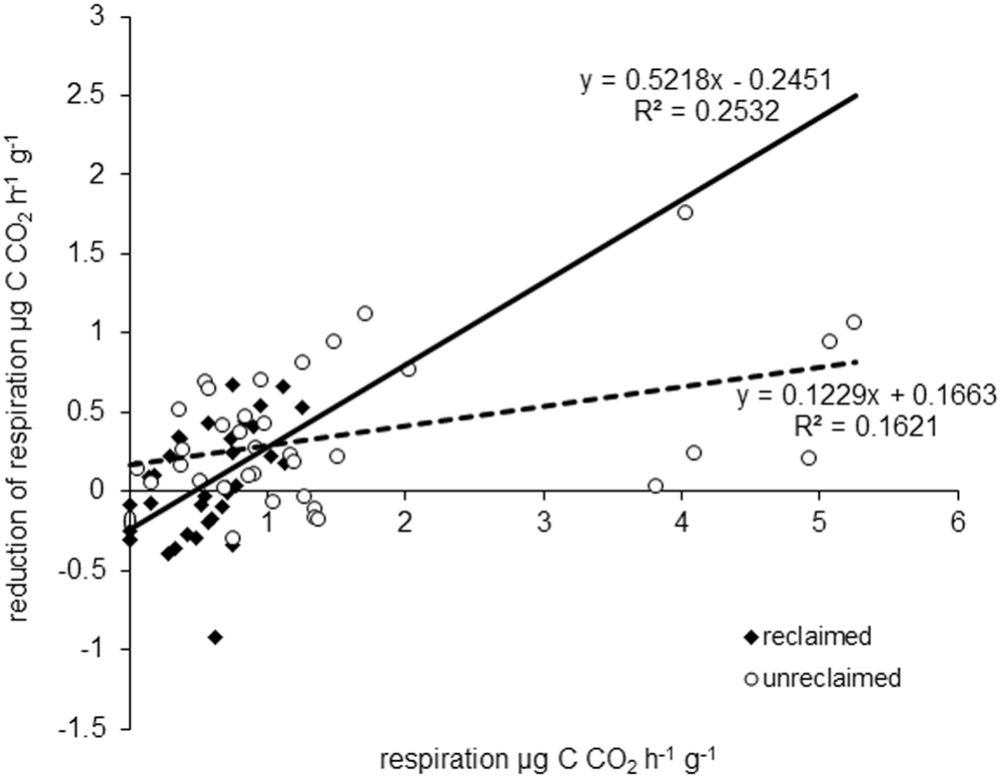
Effect of soil ventilation on soil respiration in the first laboratory experiment. The results show that a lack of ventilation reduced microbial respiration and that this reduction in microbial respiration increased as the microbial respiration in vials with ventilation increased for soil samples from both sites. Relationship between reduction of soil respiration in the simulated non-ventilation situation (on y axis) in comparison with the respiration measured in the situation when CO_2_ was continually removed (on x axis) for reclaimed and unreclaimed sites.

To assess the degree to which microbial and root respiration can be affected by ventilation of the soil, we measured the respiration in vials in which the CO_2_ either accumulated or was continually removed during incubation. For soils from both reclaimed and unreclaimed sites (whether were considered separately or together) in the first laboratory experiment, respiration was significantly lower in vials in which CO_2_ accumulated during the incubation (*t*-test, P < 0.05). When the difference in respiration between vials with and without ventilation was plotted against the respiration in vials with ventilation, the regression was significant and positive, i.e., the reduction in respiration caused by a lack of ventilation increased as respiration with ventilation increased, and this was true with soil samples from both sites (Fig. 5). In the second laboratory experiment with plant roots, in contrast, root respiration was not significantly affected by ventilation according to a *t*-test and according to GLM analysis with root biomass as a continual predictor (*data not shown*). These results indicate that microbial respiration but not root respiration is reduced when the CO_2_ concentration in the soil atmosphere is increased by microbial or root respiration.

## Discussion

In the current study we found large variation in the relationship between CO_2_ flux from the soil and CO_2_ concentration in soil. Positive relationships between CO_2_ flux from the soil and soil CO_2_ concentration are more likely in patches with higher root biomass and higher levels of CO_2_ concentration and flux. This is consistent with findings that the proportion of total soil respiration represented by root respiration is generally higher in soil locations with higher overall respiration, as indicated by a world-wide data set^6^ and by our previous study at the same sites used in the current research^20^. The latter study showed that patches with a higher root biomass are more sensitive to temperature, which may further increase the average respiration at sites with substantial levels of root respiration. The CO_2_ concentration in soil will reflect both CO_2_ production in soil and CO_2_ removal from soil pores which primarily include CO_2_ flow out of soil, but may include also other processes such as abiotic sorption of CO_2_ on water and alkali^17^ and heterotrophic CO_2_ fixation^18^. The CO_2_ flow from soil will depend on the diffusion gradient in soil and on soil aeration. Based on these simple relationships, the CO_2_ flow from the soil should be positively related to the CO_2_ concentration. This relationship may be altered, however, if soil permeability varies. If permeability decreases, CO_2_ flow will be lower at the same CO_2_ concentration. Over time, CO_2_ concentration will increase which will increase also CO_2_ flow. This effect of variable permeability can be even enhanced by a negative feedback of CO_2_ concentration on CO_2_ production, i.e., when high soil CO_2_ concentrations reduce CO_2_ production. When permeability decreases, CO_2_ flow will be lower at the same CO_2_ concentration may increase. Because an increase in CO_2_ concentration reduces CO_2_ production, this may yield in future decrease of flow this may extend periods with high CO_2_ concentration and low flow. In the extreme case of a ceasing CO_2_ production in impermeable soil, one may even have a high soil CO_2_ concentration without any CO_2_ flow from the soil. Our second laboratory experiment, which determined whether root and microbial respiration are affected by increases in CO_2_ concentration resulting from poor ventilation, indicated that root respiration was not affected by CO_2_ concentration, which is consistent with previous reports^26–28^. Although it was reported that root respiration was reduced as CO_2_ concentration increased^29^, the decrease was observed mostly at concentrations lower than usual, whereas root respiration changed only slightly at the higher concentrations common in soil. Overall, previous research indicates that common soil CO_2_ concentrations are unlikely to substantially affect root respiration.

In our first laboratory experiment, a lack of ventilation, which simulated poor soil aeration and which resulted in increasing CO_2_ concentrations, reduced microbial respiration. Moreover, the level of reduction in CO_2_ production increased with the level of microbial respiration in the vials with ventilation (Fig. 5). This observation is consistent with previous findings that microbial respiration is reduced by high concentrations of CO_2_^19,30,31^. In contrast to these earlier studies, which used CO_2_ addition, we used restricted ventilation (gas flow in the soil) to determine whether microorganisms can reduce their own respiration. One may argue that soil may not be completely sealed in the field as in our experiment, but the pores in which microorganisms live are several orders of magnitude smaller than the vials used in our experiment. Thus, we believe that a reduction in microbial respiration as a consequence of high microbial respiration and low soil aeration is likely to occur in the field. Negative relationships are likely to occur when the gas movement through the soil is very slow and the CO_2_ concentration is already high enough to substantially reduce CO_2_ production. This mechanism was previously proposed to explain the observation of negative relationships between microbial biomass and specific microbial respiration^32^. This mechanism is also consistent with our finding that negative relationships between the CO_2_ flux from the soil and soil CO_2_ concentration are correlated with high microbial biomass and respiration, low porosity, and low overall CO_2_ flux from the soil (Tables 3, 4). This may be also supported by non-random distribution of soil porosity and permeability for gases. If roots are abundant in soil, one may expect that pores in soil associated with or generated by roots will have higher connectivity and consequently will increase gas permeability more that in soil without roots. It is likely that low soil permeability for gases may be associated with spots where roots are scarce and microbial respiration dominates overall soil respiration.

**Table 4:** Summary of a multiple regression for dependent variable regression coefficients between the soil CO_2_ concentration and the CO_2_ flow from the soil at individual sampling points (as depicted in Fig. 4) and individual environmental parameters measured at these points used as predictors – regression parameters:R^2^ = 0.25738563, F_8,63_ = 2.7294, P < 0.01193.

Multiple regression parameter	Coefficient	P
absolute member	0.244	0.541
CO_2_ concentration (5–20 cm)	0.000	0.002
porosity (10–20 cm)	−1.066	0.008
respiration (g^−1^, 10–20 cm)	−0.023	0.345
bulk density (0–5 cm)	−0.196	0.561
porosity (0–5 cm)	0.051	0.170
microbial biomass (5–10 cm)	0.033	0.104
microbial biomass (0–5 cm)	−0.028	0.090
root biomass (0–20 cm)	0.005	0.292

Although the microbial production of CO_2_ can generate a negative feedback on microbial respiration, CO_2_ can also be directly metabolized by microbes^1,18^, which would remove CO_2_ from the soil air and thereby reduce the overall CO_2_ flux from the soil. A similar mechanism is well known for methane, which is produced by methanogens and consumed by methane oxidizers^16^. Microbial consumption of CO_2_ is also more likely at higher CO_2_ concentrations^18^. Both mechanisms (reduced production of CO_2_ due to negative feedback on microbial respiration and microbial consumption of CO_2_) may occur at the same time and might have occurred in our laboratory experiment, but the experimental set-up and data do not make it possible to determine which of these was more important in reducing the amount of CO_2_ in non-ventilated vials.

In addition to leaving the soil air as the result of flow into the above-ground atmosphere or microbial consumption, CO_2_ will also leave the soil air when it is dissolved in the soil water or absorbed on carbonates or other alkali in the soil^17,33–35^. However, our study sites were generated by the heaping of the same material, and our previous research shows a low variation in carbonate content and soil moisture^25,36–38^. It follows that soil water and carbonate content were unlikely to have caused the observed variation in relationship between the soil CO_2_ concentration and flux.

Another possible explanation for differing relationships in soil CO_2_ concentration and flux could be a substantial lateral movement of CO_2_ through the soil, such that the CO_2_ flows into the atmosphere not above hot spots with high CO_2_ concentration but above other locations with low CO_2_ concentration. Although this would explain low CO_2_ flow from the soil at high soil CO_2_ concentrations, it would not explain the negative relationships between flow and concentration.

Measurements of the soil CO_2_ concentration along a soil profile are often used to assess soil respiration and the contribution of individual soil depths to soil respiration^11,13,39^. In these studies, respiration is usually calculated using Fick’s first law, i.e., the calculation is based on the vertical gradient of the soil CO_2_ concentration and a gaseous diffusion coefficient of the soil CO_2_; this law assumes that diffusion is a major process that drives CO_2_ movement. Many studies have shown, however, that the relationship between diffusion and CO_2_ concentrations may be complex under field conditions. For example, the diffusion coefficient may differ substantially between depths, and as a consequence, increased CO_2_ production at shallow depths may have little effect on soil CO_2_ concentrations^34^. As already mentioned, the CO_2_ flux from soil may be affected by the amount of soil water, and a high soil CO_2_ concentration may not result in a high soil surface CO_2_ flux when soil water content is high^34^. To deal with this complexity, the latter authors proposed mathematical approaches. Moreover, after extreme events such as heavy rain, wet soil anaerobic microsites are likely to occur which may affect soil CO_2_ production; this may be affected by variation in availability of chemical compounds taking part in redox reactions such as nitrates^40^.

In conclusion, this study has shown that although the relationship between CO_2_ flux from soil and soil CO_2_ concentration is generally positive, the relationship varies from highly positive to highly negative when individual spots in the soil are assessed. The relationship tends to be positive when root biomass is high and when levels of soil CO_2_ concentration and flux are high. The relationship tends to be negative when microbial biomass and microbial respiration are high and when soil porosity is low. Laboratory experiments indicated that microbial respiration but not root respiration may be reduced by a rising soil CO_2_ concentration when “ventilation” (aeration or gas flow through soil) is limited. We therefore propose that negative relationships between CO_2_ flux from soil and soil CO_2_ concentration may be caused by variation in gas permeability and enhanced by a negative feedback between the soil CO_2_ concentration and microbial respiration.

## Methods

### Field study

A field study was done at the Podkrušnohorská heap, which was produced by open-cast coal mining near Sokolov in the Czech Republic (50°14′21″N, 12°39′24″E), altitude 500–600 m a.s.l. The mean annual precipitation is 650 mm, and the median annual temperature is 6.8 °C. The spoil heap consisted of tertiary clay shales^21,22^. The pH of the substrate is alkaline in initial successional stages and gradually decreases with site age. The soils are well aerated upland soils with no standing water in soil profile. We used one unreclaimed and one reclaimed site, both of which were 25–30 years old. The site age was calculated since the last major disturbance, which was a heaping for the unreclaimed site and a leveling for the reclaimed site. The reclaimed site was planted with alder (*Alnus glutinosa* and *Alnus incana*), which is the most common reclamation measure in the area. The reclaimed site had a canopy formed by closely spaces trees that were 6–8 m tall^23^. The unreclaimed site has been spontaneously colonized by plants and especially by *Salix caprea*, *Betula pendula*, and *Populus tremula*. Vegetation develops more slowly at unreclaimed than reclaimed sites; the canopy closes in about 10-years-old sites at reclaimed sites and in about 20-year-old sites in unreclaimed sites^23^. No topsoil was applied at the reclaimed or unreclaimed site, and the initial soil profile had already formed at both sites^21,24^. The reclaimed site had been leveled by earthmoving machinery before trees were planted. No such procedure was carried out at the unreclaimed site, which like other unreclaimed sites, was characterized by longitudinal rows of depressions and elevations, or “waves”, formed during the heaping process. The top of a wave was about 1–2 m above the bottom of the depression, and individual waves were about 6 m apart. Waves generate a variation in the plant community as well as in many soil properties^25^. See supplementary 1 for key soil properties based on previous studies^20,38^.

### Field measurements of soil respiration and soil CO_2_ concentration and soil analyses

Measurements were made during the first week of each month during the growing season in 2011 (from July to September) and 2012 (from May to September), yielding a total of 9 sampling times. In total, 72 permanent measurement points were established, 36 at the unreclaimed site and 36 at the reclaimed site. Respiration at the soil surface (i.e., the quantity of CO_2_ moving from the soil to the atmosphere) was measured *in situ* with an SR1000 CO_2_ analyzer (ADC UK©) (Fig. 1). This instrument works as an open system that uses an infrared CO_2_ analyzer to measure the CO_2_ concentration in air entering and leaving a respiration chamber, together with air flow through the chamber. During respiration measurement, soil temperature was recorded at 2 cm depth with a temperature sensor that was integrated into the SR1000 gas analyzer. For each sampling point, three readings 20 s apart were taken and averaged. At the same time and at the same point, we measured the soil CO_2_ concentration using two Vaisala sensors located in a perforated tube (Fig. 1). One tube was positioned horizontally at a depth of 5 cm, and the other was positioned vertically with openings at a depth of 5–20 cm. At each point and sampling time, we therefore obtained a measurement of CO_2_ flux at the soil surface and CO_2_ concentration at two spots (one at 5 cm depth and one at 5–20 cm depth). The order in which points were sampled was randomized at each sampling time.

After the two seasons of field respiration measurements, two soil cores (11 cm in diameter and 20 cm deep) were taken from each measurement point and were divided into three layers (0–5, 5–10, and 10–20 cm). After the roots were removed, the soil in the first core used to measure microbial respiration and biomass and was also used for the first laboratory incubation experiment (see the next section); the roots were placed on a sieve, washed with water, dried, and weighed. The second core was dried at 60 °C for 24 h and was used to measure bulk density, soil density, and pH.

To assess microbial respiration in each layer of each sampling point, 10 g of fresh soil was placed in hermetically sealed 100-mL vials (Fig. 2). CO_2_ produced in the vials was trapped in 3 mL of 1 M NaOH. Vials were kept at 20 °C for 7 days; the amount of trapped CO_2_ was determined by HCl titration after the addition of BaCl_2_. Blank samples without soil were used to correct for the CO_2_ that entered the vials with air during handling. Microbial biomass was measured by the chloroform-fumigation and extraction method^41^. Bulk density was measured by weighing the whole layer of the core after drying at 60 °C for 48 h. Soil density was established with a pycnometer^42^. Bulk density and soil density were then used to calculate soil porosity^27^. pH (in H_2_O 1:5 w/v ratio) was measured with a glass electrode.

### Laboratory experiments

The first laboratory experiment was conducted to assess the effect of reduced ventilation on microbial respiration. The set-up was similar to that used for measuring respiration in the previous section, i.e., 10 g of fresh soil (from each of three layers of a soil core collected at each sampling point) was placed in hermetically sealed vials, and the CO_2_ produced in each vial was trapped in 3 mL of 1 M NaOH. For each sample, however, two sets of vials were prepared in three replicates. In the first set of vials, NaOH was added at the beginning of the experiment. In the second set of vials, an empty beaker was placed in the vial, and the same amount of NaOH was added by a syringe and needle through a septum 1 day before the end of the experiment (Fig. 2). Vials were kept at 20 °C, and after 7 days the amount of the trapped CO_2_ was determined by HCl titration after the addition of BaCl_2_. In the first set of vials, the partial concentration of CO_2_ was close to zero during the whole incubation because the CO_2_ was continually removed by NaOH. In the second set of vials, the CO_2_ concentration increased during the incubation and mimicked a situation in which the CO_2_ concentration increases in soil pores because of limited ventilation (limited permeability of soil for gases). Blank samples without soil were established for both sets of vials; CO_2_ concentrations did not significantly differ between the blank samples in the first and second set.

A similar set-up was used in the second laboratory experiment to measure the effect of reduced ventilation on root respiration. To place the roots in the vials, spruce (*Picea abies*) seedlings were fixed in a rubber septum using silicone such that the aboveground part of the seedling was in contact with the air outside of the vial, and the roots, which were in sand packed in nylon mesh (0.1 mm), were in the hermetically sealed vial (Fig. 2). Spruce seedlings were used because they were abundant in the understory of the field sites^21^ and because we were able to obtain sand-grown seedlings from a nursery. The seedlings in the nursery were 9–12 cm tall (excluding roots) and had a stem diameter at the base of about 1.5 mm. The seedlings were removed from the nursery along with the sand surrounding the roots, and the roots and surrounding sand were immediately wrapped in the nylon mesh and transported to the laboratory. Before the experiment, seedlings were arranged according to size and then divided into two sets so that each set contained seedlings of about the same size. As in the first laboratory experiment, two sets of vials were established, i.e., NaOH was added at the beginning of the experiment to one set and 1 day before the end of the experiment to the second set. Each set was represented by 12 replicate vials. At the end of the experiment, the roots were separated from the seedlings, washed, dried at 40 °C for 12 h, and weighed.

### Data processing and analyses

Linear and logarithmic regression were used to explore relationships between the CO_2_ flow from the soil and the soil CO_2_ concentration at each of the two depths and between the CO_2_ flow from the soil and the differences in soil CO_2_ concentration between the two depths; these regressions were done across the two sites, i.e., with the pooled data from both the reclaimed and unreclaimed site. Correlation was used to explore relationships between environmental parameters measured at individual sampling points and the CO_2_ flow from the soil, the soil CO_2_ concentration at each depth, and the differences in soil CO_2_ concentration between depths at each sampling point; this was done across the sites and for each site separately. Except for temperature, which was measured at all sampling times, environmental parameters were measured only once after the last field measurement. Multiple regression with forward selection was used to predict the CO_2_ flow from the soil and the CO_2_ concentration in the soil based on the environmental parameters.

In addition to relationship described above which combine spatial and temporal variation, we also study relationship between CO_2_ flows and soil CO_2_ concentrations in the 5–20 cm depth using temporal variation in those data at each individual sampling points. Only the 5–20 cm depth was used as both depths as well as between-depth difference were highly correlated. These relationships obtained at individual sampling points were highly variable. To explain pattern of this variation the correlation coefficient and slopes of the linear regressions obtained between the CO_2_ flow and the CO_2_ concentration in the 5–20 cm depth for individual points were then correlated with the values for environmental parameters obtained for these points. The correlation coefficient of the linear regression obtained between the CO_2_ flow and the CO_2_ concentration in the 5–20 cm depth was also subjected to multiple regression to determine how this correlation coefficient was explained by other environmental parameters obtained at these points.

To evaluate the effect of ventilation in the first laboratory experiment, we used a paired *t*-test that compared vials to which NaOH was added at the beginning vs. at the end of the experiment. In the second laboratory experiment, we used a normal *t*-test to compare data from the two kinds of vials. We also used the GLM in which the NaOH treatment was a fixed predictor and the root biomass was a continual predictor. In addition, we calculated the linear regression between the reduction of microbial respiration in vials to which NaOH was added at the end (calculated as the difference between the respiration in vials to which NaOH was added at the beginning vs. at the end) and respiration in vials to which NaOH was added at the beginning. All computations were done using Statistica 13.0. Values are presented as means ± standard deviation. All regression lines presented are significant at P < 0.05.

### Data Accessibility

All data are uploaded as online information using Dryad Digital Repository 10.5061/dryad.41sk145.

## Electronic supplementary material

Supplementary S1
