# Supplementary material for: Flow of CO2 from soil may not correspond with CO2 concentration in soil
Source: Sci Rep. 2018 Jul 4;8:10099. doi: 10.1038/s41598-018-28225-z (PMC6031679; doi:10.1038/s41598-018-28225-z)
Supplement: Supplementary file 1 — Supplementary S1 [file 41598_2018_28225_MOESM1_ESM.docx]

**Supplementary information**

**Flow of CO_2_ from soil may not correspond with CO_2_ concentration in soil**

Jan Frouz & Luděk Bujalský

^1^Institute for Environmental Studies, Faculty of Sciences & Environmental Centre, Charles University, Benátská 2, 12800, Prague, Czech Republic

Supplementary S1: Key soil characteristic of investigated plots based on previous studies ^20,39^.

| Parameter (unit) | Alder plantation | Succession depressions | Succession elevations |
| --- | --- | --- | --- |
| Bulk density (g cm^-3^) | 0.73±0.08 | 0.93±0.08 | 0.83±0.06 |
| Porosity (% volume) | 67±4 | 61±5 | 53±4 |
| Water field capacity (% volume) | 57±3 | 55±5 | 51±4 |
| Clay content (%) | 27±6 | 13±1 | 17±2 |
| pH | 7.5±0.2 | 7.6±0.3 | 7.5±0.2 |
| C (%) | 9.8±1.9 | 11.5±8.1 | 5.9±2.3 |
| Root biomass (g m^-2^) | 115±34 | 229±18 | 33±7 |
